# Study on Mechanical Behaviours and Microstructure Features of Q690 Steel Weldments with Various Electrochemical Hydrogen Charging Conditions

**DOI:** 10.3390/ma17225446

**Published:** 2024-11-07

**Authors:** Wen-Jiao Dan, Cheng-Wang Tang, Hao Shi, Xu-Yang Wang

**Affiliations:** 1 School of Mechanical Engineering, Anhui Science and Technology University, Chuzhou 233100, China; 2 School of Ocean & Civil Engineering, Shanghai Jiao Tong University, Shanghai 200240, China

**Keywords:** high-strength steel, welded components, mechanical behaviour, electrochemical hydrogenation, HAZ

## Abstract

Regarding the application of high-strength steel welded components to large marine equipment, prolonged exposure to marine environments results in the infiltration of hydrogen, leading to a significant decline in mechanical performance. In this study, the electrochemical hydrogenation characteristics of Q690 high-strength steel welded components at room temperature are examined under various conditions to investigate their mechanical properties. The welded specimens initially undergo electrochemical hydrogenation and, subsequently, uniaxial tensile testing to explore the influences of hydrogenation methods on their mechanical properties such as tensile strength, yield strength, and elongation after fracture. X-ray diffraction (XRD) and scanning electron microscopy (SEM) are utilized to observe changes in the microstructure features in heat-affected zones (HAZs) and highlight the mechanisms affecting material mechanical properties. The results indicate that the tensile strength, yield strength, and elongation after fracture of the material decrease with increasing hydrogenation time, solution concentration, and current density. Moreover, the fracture modes of Q690 high-strength steel welded components shift from ductile fracture to brittle fracture with increasing hydrogen atom penetration.

## 1. Introduction

With the advancement of modern science and technology, the utilization of land resources has become insufficient to satisfy societal demands, prompting a gradual shift towards marine resources. Concurrently, there is an increasing demand for high-strength steel weldments in the fabrication of marine equipment [1]. Nevertheless, the intricate working conditions within marine environments pose challenges for high-strength steel welding. Exposure to seawater in humid environments during service leads to the infiltration of hydrogen molecules into the interior of high-strength steel weldments, consequently inducing brittleness or cracking in the metal material [2,3].

Previously, researchers have investigated the impacts of hydrogen-induced damage on the performance characteristics of high-strength steel weldments. Zielinski et al. [4] conducted a study evaluating the susceptibility levels of Q690 high-strength steels and its welded joints to hydrogen embrittlement in marine environments. Similarly, Wei et al. [5] examined the corrosion behaviour and time-dependent effects of butt-welded joints of Q690 high-strength steel in marine environments. In the examination of the hydrogen damage behaviours of high-strength steel weldments across various welding methods, Ćwiek et al. [6] concluded that these weldments exhibit pronounced susceptibility to hydrogen embrittlement when subjected to increasing welding current during hydrogen charging. Yu et al. [7] developed a model for assessing the mechanical properties and microstructures of coarse-grain HAZs in high-strength, low-alloy steels. Garrison Jr. et al. [8] experimentally investigated hydrogen embrittlement in high-strength steels by examining six steels with different microstructures. Moreover, Song et al. [9] studied stress corrosion cracking in X100-grade pipeline steel immersed in carbonate/bicarbonate solution. Numerous scholars have investigated the hydrogen-induced damage behaviours of high-strength steel weldments resulting from various pre-welding treatments. Wang et al. [10] examined the hydrogen-induced cracking threshold stress intensity (KIH), crack extension rate (da/dt), and hydrogen diffusion coefficient (D) of 30CrMnSiNi2 steel. Li et al. [11] explored the impact of shear pre-strain on hydrogen embrittlement by observing the characteristics of intergranular fracture on the specimen surface during tensile tests with in situ hydrogen charging, which decreases the ductility of the material, particularly after hydrogen charging. Zheng et al. [12] investigated the hydrogen embrittlement properties of manganese aluminium bainitic steel used in railway tracks and noted a decrease in hydrogen embrittlement severity with increasing aluminium content in the steel. Okayasu et al. [13] investigated the hydrogen embrittlement properties of high-strength steel plates with varying microstructural characteristics. Gong et al. [14] scrutinized the embrittlement mechanism of advanced high-strength steels, observing that hydrogen charging increases the dislocation densities and strain fields surrounding steel precipitates, consequently increasing residual stresses.

In diverse hydrogen charging experiments, Venezuela et al. [15] investigated the impacts of hydrogen on martensitic advanced high-strength steels under cathodic hydrogen charging conditions. Drexler et al. [16] addressed the issues of hydrogen embrittlement in high-strength steel plates, noting the minimal hydrogen content in the bulk material and the exponential increases in the hydrogen content at the microscale shear affected zone with severe plastic deformation. Meda et al. [17] observed a heightened susceptibility to hydrogen embrittlement in high-strength steels with increased steel strength, as evidenced by studies of materials used in hydrogen storage cylinders. By manipulating the microstructure, Park et al. [18] investigated hydrogen embrittlement in ultrahigh-strength steels with tensile strengths surpassing 1500 MPa. MoroI et al. [19] explored the hydrogen embrittlement susceptibility of high-strength steel X80 and noted various types of damage induced by hydrogen, including debonding along the ferrite/pearlitic interface and microcrack formation on the outer surface of the specimen. Ma et al. [20] investigated hydrogen-induced blistering in Q690 high-strength steel under different hydrogen charging conditions. Toji et al. [21] studied 1180 MPa cold-rolled duplex steel plates and identified factors contributing to hydrogen embrittlement in high-strength steels, emphasizing the contribution of plastic strain induced by cold working, the external application of stress, and the amount of diffusible hydrogen entering the steel. Lovicu et al. [22] examined hydrogen embrittlement in four advanced high-strength steels, linking hydrogen embrittlement susceptibility to strength and microstructural characteristics after electrochemical hydrogen charging. Allen et al. [23] conducted hydrogen charging and tensile experiments on two advanced high-strength steel types, analyzing microcrack patterns and hydrogen-induced pore changes on material surfaces. In summary, the electrochemical hydrogenation method and hydrogenation conditions significantly impact the material properties of high-strength steel weldments. With the permeation of hydrogen molecules, the tensile strength and elongation of high-strength steel gradually decrease. Hydrogen molecules change the microstructure of high-strength steel, causing changes in crystal shape and microstructure. Therefore, certain changes in the fracture morphology of the material occur, and a transition from initial ductile fracture dimples to transgranular fracture arises, thereby resulting in hydrogen-induced brittle fracture phenomena.

In this study, Q690 structural high-strength steel weldments were chosen. The aim was to explore the mechanical properties of the materials by following hydrogen-induced damage using various electrochemical hydrogen charging conditions with uniaxial tensile testing, X-ray diffraction analysis, and microstructural examination. The degradation mechanical parameters with the various hydrogen charging methods (such as the tensile strength, yield strength, and elongation after fracture of the material) were analyzed. The microstrain and dislocation density of the HAZ of the welding specimen were calculated by using the results of the X-ray tested, and the fracture morphology and microstructure in the HAZ were also evaluated.

## 2. Materials and Methods

**Figure 1:**
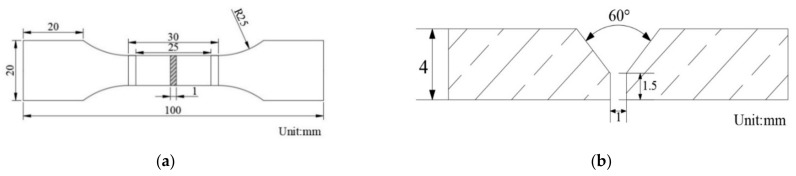
Schematic diagram of the specimen’s (**a**) size (mm) and (**b**) welding bevel schematic.

In this study, Q690 high-strength steel welded specimens were utilized, each comprising a plate piece with a total length of 100 mm and a thickness of 4 mm. The chemical composition of the sample is shown in Table 1. A center section of 25 mm in length was designated for electrochemical hydrogen charging experiments, as shown in Figure 1a, and a schematic view of the weld bevel is shown in Figure 1b.

The welding positions of the specimens were prepared with a 60° V type bevel, with a depth of 2.5 mm, as illustrated in Figure 1b. The welding process employed carbon dioxide gas-shielded welding, with a gas mixture consisting of 80% Ar and 20% CO_2_. The welding parameters were set to a current of 200 A and a voltage of 22 V. An MG80-G wire that was compliant with the GB/T39281 [24] specification was utilized for welding. This wire, which was categorized as a mixed-gas-shielded solid wire for high-strength steels of the Gr-Ni-Mn-Ti type, had a diameter of 1.2 mm. The chemical composition of the welding wire is detailed in Table 2.

**Table 1:** Q690 high-strength steel chemical composition table (mass fraction %) [25].

Chem. Comp.	C	Mn	Si	P	S	Cr	Ni	Mo	Nb
value	0.17	1.38	0.41	0.22	0.18	1.12	1.38	0.54	0.10

**Figure 2:**
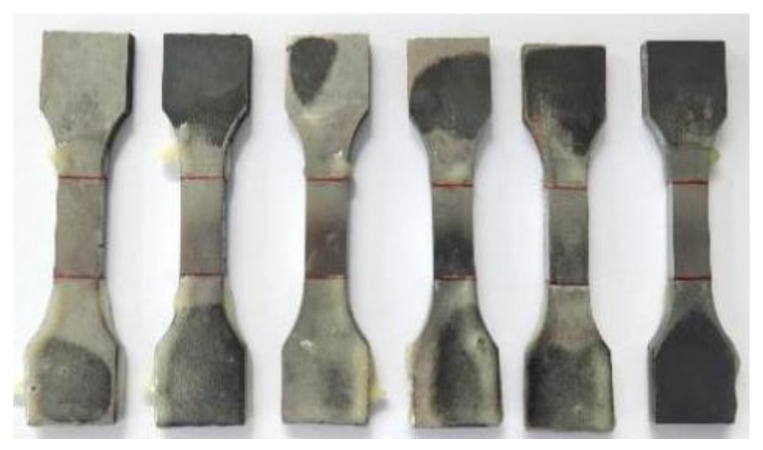
Q690 high-strength steel weldment specimens (the area between the two red lines of the specimen is the part for the electrochemical hydrogen charging).

First, the high-strength steel weldments were ground with #2000 silicon carbide sandpaper layer by layer, polished, rinsed with alcohol, and then dried in cold air. The pre-hydrogenated surface had a length of 25 mm, a width of 10 mm, and a hydrogenated area of 2.5 cm^2^, and the remaining specimen was wrapped with epoxy resin coating, as shown in Figure 2.

**Figure 3:**
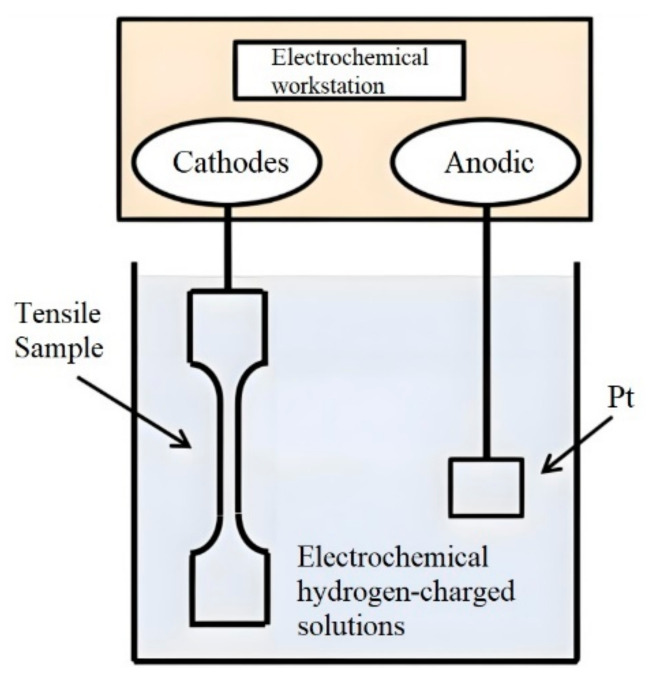
Schematic diagram of electrochemical hydrogen charging.

The specimen was connected to the cathode, a platinum sheet was used as the anode, and constant-current electrochemical hydrogen charging was performed on the Q690 high-strength steel weldments by adding 1 g/L, 5 g/L, or 10 g/L CH4N2S solution to H_2_SO_4_ at a concentration of 0.5 M/L. A schematic diagram of the hydrogen charging experimental assembly is shown in Figure 3.

Three groups of hydrogen charging experiments were used for electrochemical hydrogen charging, and the specific hydrogen charging conditions used are shown in Table 3.

A block measuring 10 mm × 10 mm × 4 mm was excised from the central position of the hydrogen-charging specimen and meticulously polished using sandpaper to eliminate surface oxides and other non-metallic impurities. The dislocation density in the HAZ of Q690 high-strength steel weldments after hydrogen charging was qualitatively evaluated using a Shimadzu XRD-6100 X-ray diffractometer. The X-ray generator was configured with a Cu target operating at a tube voltage of 35 kV and a tube current of 40 mA. The testing included angles ranging from 30° to 90° at a scan rate of 20 deg min^−1^ [26]. After testing, the X-ray diffraction (XRD) data were analysed via Jade 6.5 software, and the dislocation density was determined via the Williamson–Hall method. Introduced by Williamson and Hall in the 1950s, the Williamson–Hall (WH) method could be used to characterize changes in the dislocation densities of materials by modelling the broadening of the diffraction peak induced by microstructures within crystals [27].

The specimens were subjected to a unidirectional tensile test at room temperature immediately after hydrogen charging, and the entire tensile test was completed within twenty minutes to prevent the leakage of hydrogen from the material. The equipment used for the experiments was a Sansi UT5105 electronic universal testing machine, (Sansi, Shanghai, China), with a maximum tensile force of 100 kN and a tensile speed of 3 mm/min. The number of specimens used in the test is 3 per sample to ensure the accuracy of the data.

The Q690 high-strength steel welded specimens were polished with #2000 sandpaper. Then, these samples were subjected to electrochemical hydrogen charging experiments. Subsequently, the specimens were polished and corroded using a 4% nitric acid alcohol corrosion solution. After the experiments, a 5 mm × 5 mm × 4 mm specimen was cut in the center of the specimen, and the heat-affected zone of the weldment was analyzed by scanning electron microscope (SEM) with a Zeiss EVO18 scanning electron microscope.

## 3. Results

This section discusses the experimental results of the variation in mechanical properties, microstructure, and fracture form in the HAZ of welded specimens of Q690 high-strength steel with different hydrogen charging variables.

### 3.1. Effect of Hydrogen Charging Conditions on the Mechanical Behaviours of Weldment Specimen

**Figure 4:**
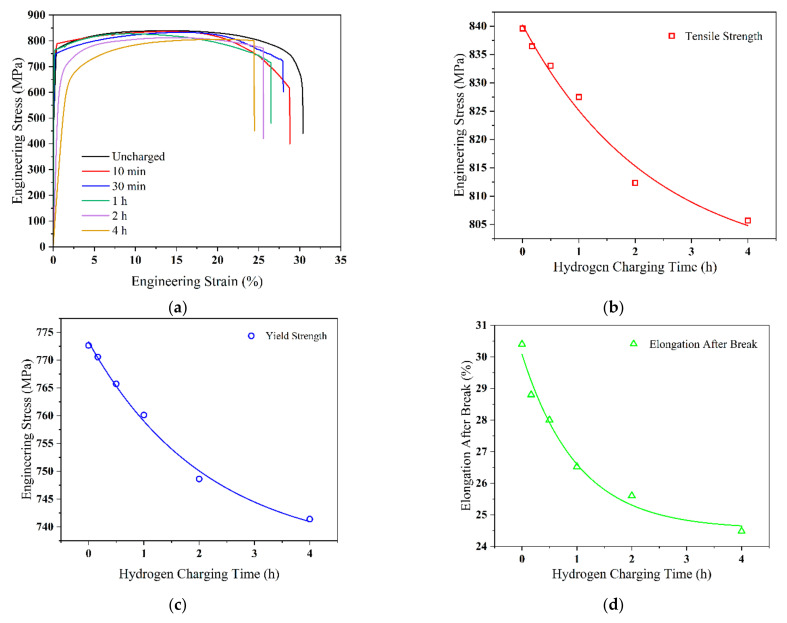
Tensile and mechanical properties of Q690 high-strength steel weldments with different hydrogen charging times. (**a**) Stress–strain curves; (**b**–**d**) tensile strength, yield strength, and elongation vs. hydrogen charging time, respectively.

The tensile stress–strain curves of the Q690 high-strength steel weldments after various hydrogen charging times are shown in Figure 4a.

The results indicate that extension of the curves gradually decreases with increasing hydrogen charging time, although the change before the necking point is minimal. Both the tensile and yield strengths of the material gradually decrease, with the tensile strength decreasing from the initial value of 793 MPa to 710 MPa. In weldments, yielding phenomena are typically less pronounced during tensile experiments [28]. The occurrence of hydrogen embrittlement significantly affects the yield characteristics of weldments, leading to the loss of a well-defined yield point and a reduction in plasticity, which, in turn, increases the brittleness of the material. In tensile tests, welds influenced by hydrogen are more prone to abrupt fracture, rather than displaying a distinct yielding phase. The curves for a hydrogen charging time of 10 min closely resemble those for 30 min, with little difference in tensile strength and a decrease in elongation. However, the elongation of the material rapidly decreases after 1 h of charging. This phenomenon is attributed to the gradual penetration of hydrogen molecules into the material over time, which disrupts the crystallinity, thereby increasing grain boundary margins and reducing elongation. The elongation decreases from 19.2% to 13.2% when the hydrogen charging time increases to 1 h and further decreases to 9.8% when the hydrogen charging time increases to 4 h.

Figure 4b–d display the fitted data plots of the tensile strength, yield strength, and elongation at different hydrogen charging times, respectively. The decreasing trend of the fitted functions indicates that the tensile strength, yield strength, and elongation of the materials sequentially decrease. Thus, the hydrogen charging time significantly influences the hydrogen embrittlement properties of the material, leading to a decrease in its mechanical tensile properties.

**Figure 5:**
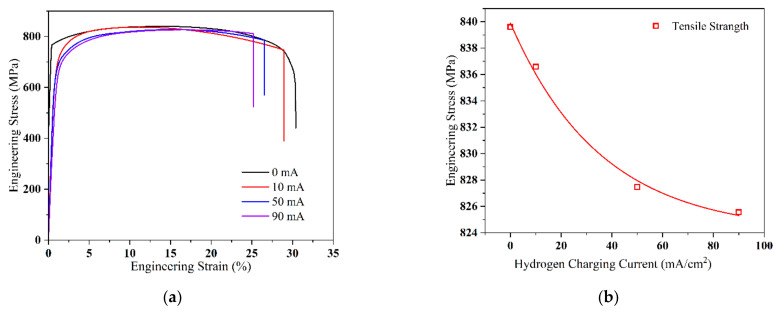

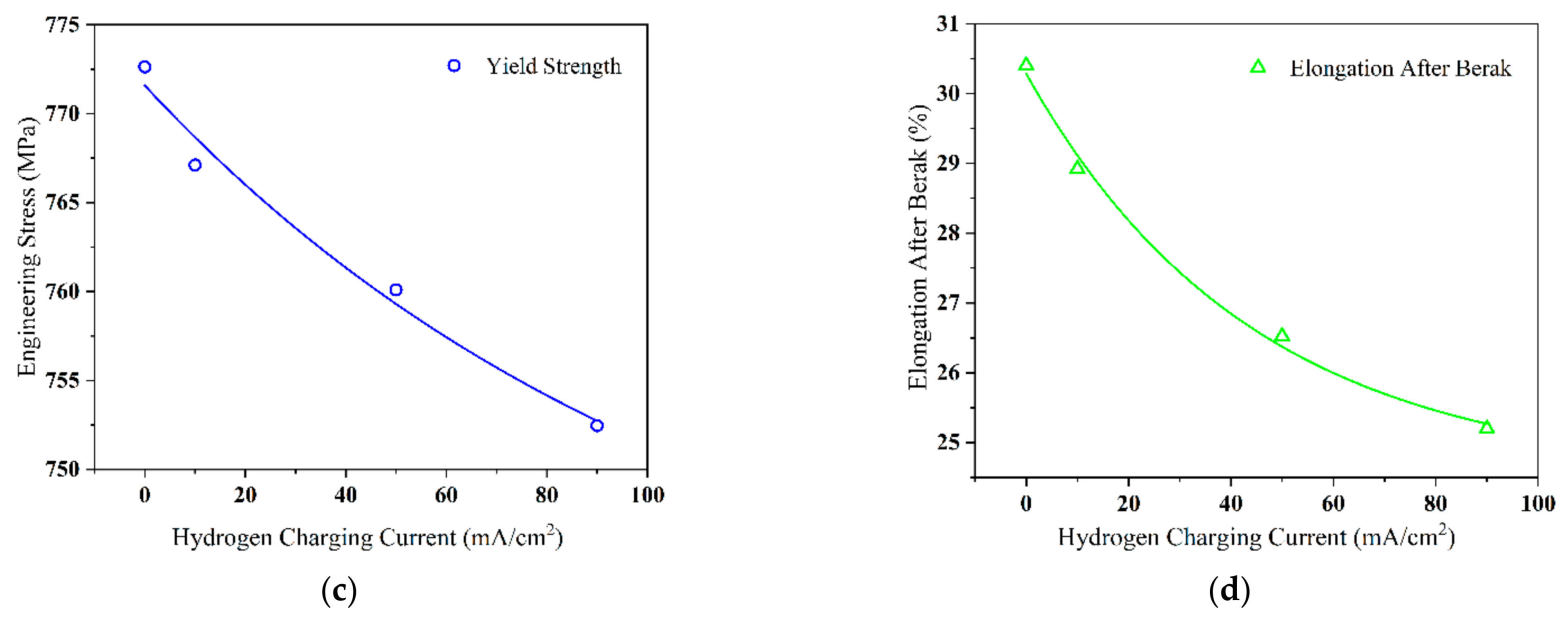
Tensile mechanical properties of Q690 high-strength steel weldments with different hydrogen charging current densities. (**a**) Stress–strain curves; (**b**–**d**) tensile strength, yield strength, and elongation vs. hydrogen charging current densities, respectively.

The stress–strain curves of the Q690 high-strength steel weldments, subjected to different hydrogen charging current densities, are displayed in Figure 5a.

Evidently, the tensile and yield strengths of the material exhibit opposing decreasing trends as the hydrogen charging current density increases. Figure 5b–d show the fitting curves of the tensile strength, yield strength, and elongation after fracture, respectively. Notably, as the current density increases from 10 mA/cm^2^ to 90 mA/cm^2^, the tensile strength and elongation of the material decrease linearly. The significant change in elongation of the specimen is attributed to the gradual decline in the mechanical properties of the high-strength steel due to continuous hydrogen atom penetration.

**Figure 6:**
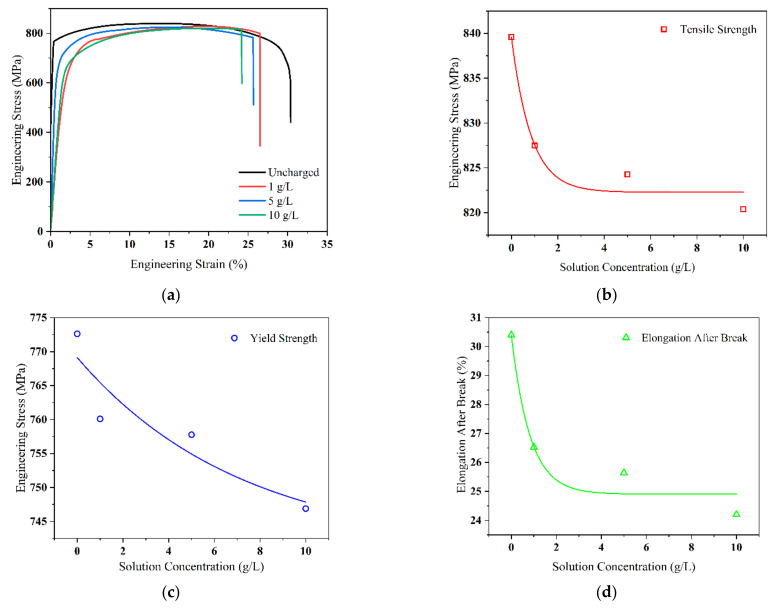
Tensile mechanical properties of Q690 high-strength steel weldments with different hydrogen charging solution concentrations. (**a**) Stress–strain curves; (**b**–**d**) tensile strength, yield strength, and elongation vs. hydrogen charging solution concentrations, respectively.

Figure 6a shows the tensile stress–strain curves of Q690 high-strength steels at various concentrations, revealing significant decreases in the overall strength and elongation parameters when the solution concentration reaches 1 g/L.

The elongation of the material sharply decreases with further increases in solution concentration to 5 g/L, while the tensile strength and elongation reach their minimum values as the concentration continues to increase to 10 g/L. Figure 6b–d depict plots of the fitted data for the tensile strength, yield strength, and elongation of the material, respectively.

**Figure 7:**
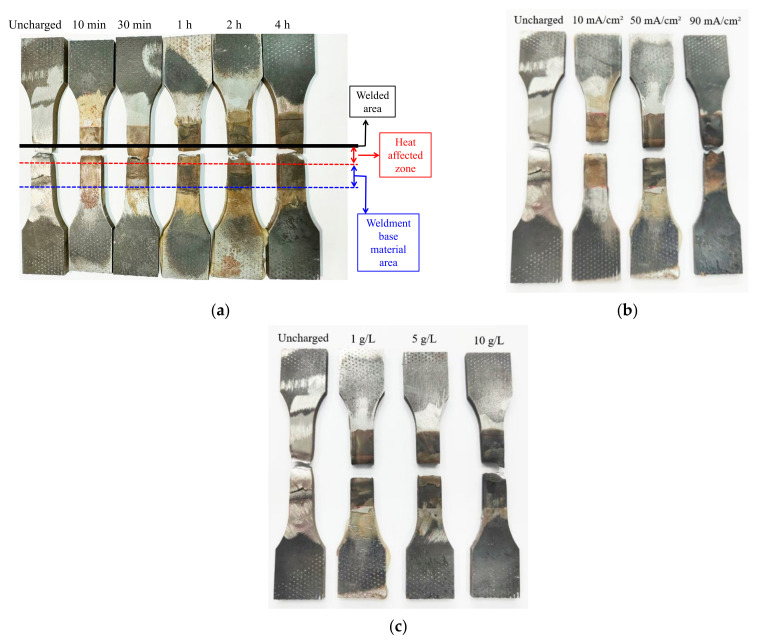
Q690 high-strength steel weldments after pull-off with different hydrogen charging conditions. (**a**) Hydrogen charging times; (**b**) current densities; (**c**) solution concentrations.

The pull-off specimens after different electrochemical hydrogen charging experiments are shown in Figure 7.

The depth of the corrosion color ranges from light to dark on the pull-off specimens at different hydrogen charging parameters. The black solid line represents the weld zone, the distance between the black solid line and the red dashed line represents the heat-affected zone, and the distance between the red dashed line and the blue dashed line represents the base material zone of the weldment (Figure 7a).

Mechanical parameters and corresponding residual coefficients of welded parts of Q690 high-strength steel, with different hydrogen charging time, current density, and solution concentration, are shown in Table 4, Table 5 and Table 6, in which mechanical parameters $\sigma_{b}$, $\sigma_{s}$, and *δ* are the strength stress, yield stress, and elongation after fracture of the material after different hydrogen charging conditions, respectively, and $\sigma_{b0}$, $\sigma_{s0}$, and $\delta_{0}$ represent the corresponding values of the material uncharged, which are 839.59 MPa, 772.63 MPa, and 30.40%, respectively.

Under three distinct electrochemical hydrogen charging conditions, the tensile strength of the material decreases by approximately 1–4%, the yield strength decreases by about 2–4%, and the elongation at fracture decreases by roughly 17–20%. These results indicate that while the hydrogen charging conditions have a minor impact on the material’s strength, they significantly affect its elongation.

**Figure 8:**
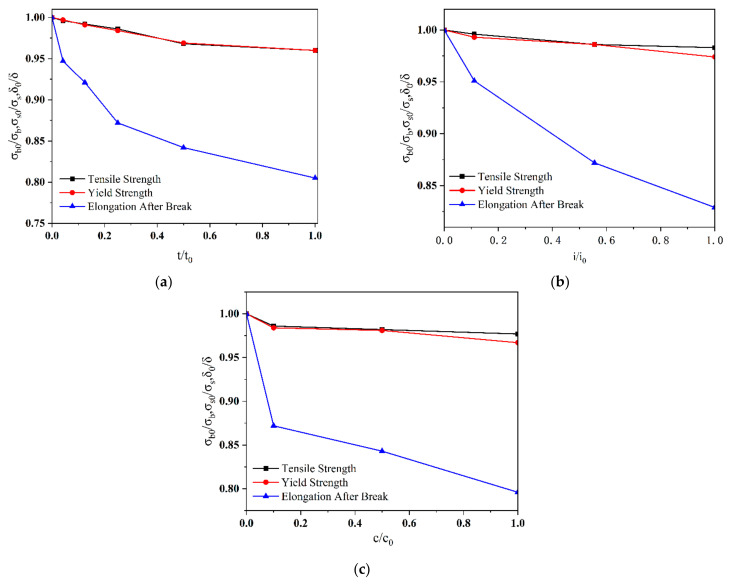
Normalized mechanical parameters of Q690 steels with various hydrogen charging methods. (**a**) Normalized mechanical parameters vs. hydrogen charging time; (**b**) normalized mechanical parameters vs. current density; (**c**) normalized mechanical parameters vs. solution concentration.

The results of residual coefficients for the welded parts of Q690 high-strength steel indicated that $\delta /\delta_{0}$ is about 0.956, 0.950, and 0.944 as the hydrogen charging time is 4 h for No. 1 (in Table 4), the hydrogen charging current density is 90 mA/cm^2^ for No. 2 (in Table 5), and CH4N2S solution concentration is 10 g/L for No. 3 (in Table 6). To comparatively assess the impacts of hydrogen charging conditions on the mechanical characteristics of the Q690 high-strength steel, the hydrogen charging parameters (the hydrogen charging current density *i*, hydrogen charging time *t*, and hydrogen charging solution concentration *c*) are also normalized, and the reference values $i_{0}$, $t_{0}$, and $c_{0}$ are 4 h, 90 mA/cm^2^, and 10 g/L, respectively. The results demonstrated a non-linear decrease in residual factors of the tensile strength, yield stress, and elongation with increasing normalised hydrogen charging parameters. The rate of decrease in the residual factors of the tensile strength and yield stress is much lower than that in the elongation. Moreover, the variation in the residual factors concerning tensile strength and yield stress are similar (in Figure 8).

**Figure 9:**
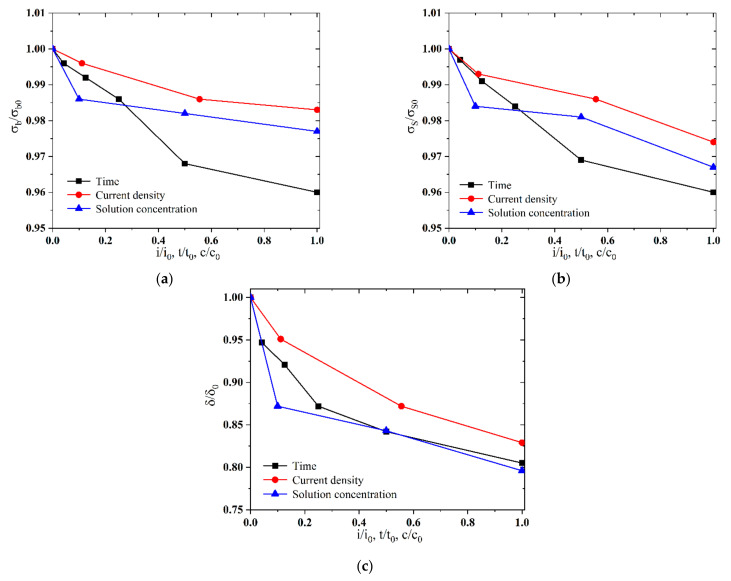
Normalized mechanical parameter vs. normalized hydrogen charging parameters. (**a**) Normalized tensile strength; (**b**) normalized yield strength; (**c**) normalized elongation at break.

As the hydrogen charging time/solution concentration increases, the degradation rate of the mechanical parameters decreases. Compared with the three hydrogen charging conditions, the hydrogen charging current density exerts the least impact on the degradation of mechanical parameters of materials. Initially, the duration of the hydrogen charging solution significantly affects the degradation of mechanical parameters. However, in the middle and later stages, the concentration of the hydrogen charging time has a more pronounced influence on the degradation of mechanical parameters (in Figure 9).

### 3.2. Effect of Hydrogen Charging Conditions on the Micro-Features in the HAZ

Due to the cyclic heat effect in the welding process, coarse ferrite and fine pearlite may appear locally in the HAZ of the welded part, leading to non-uniform distribution of its microstructure and a decrease in the mechanical behaviours of the HAZ. In addition, the mechanical properties of the HAZ further decrease after hydrogen charging. In our study, the experimental XRD diffraction method was used to study the influence of hydrogen charging on the microstrain and dislocation density in the HAZ.

**Figure 10:**
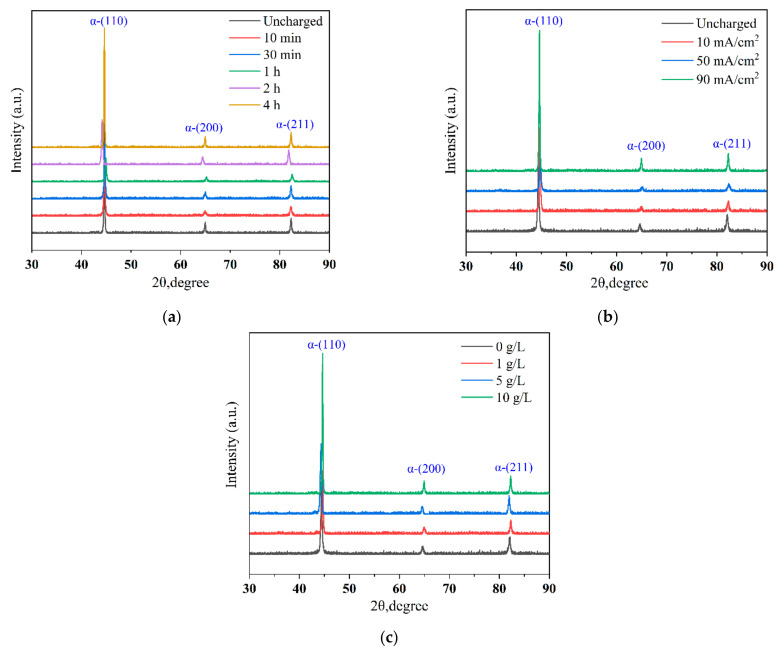
XRD patterns of HAZ of Q690 high-strength steel weldments at various hydrogen charging conditions. (**a**) Time; (**b**) current density; (**c**) solution concentration.

The experimental results (in Figure 10) show that the heights of the diffraction peaks increase sequentially over parameters (such as time, current density, and solution concentration).

Hydrogen penetration and diffusion can lead to significant changes in the microstructure of materials, including lattice expansion and an increased dislocation density, which facilitate the formation and growth of ferrite phases. The diffusion of hydrogen atoms creates additional nucleation sites, thereby promoting more rapid ferrite growth. Furthermore, hydrogen can influence the stability of various phases by altering the interfacial energy or chemical potential between them. Under certain conditions, hydrogen penetration can enhance the stability of the ferrite phase relative to other phases, such as austenite, resulting in a higher proportion of ferrite [29]. The diffraction peak heights in Figure 10 reflect the entry of hydrogen molecules into the specimen during cathodic hydrogen charging. The increasing hydrogen content in the specimen during these experiments induces compressive stresses and microstructural changes on the surface. When hydrogen atoms enter the specimen, they expand the metal lattice, causing internal tension and material deformation, which increases with an increase in the hydrogen charging parameter [30]. The peak diffraction value indicates the diffraction intensity of the material, which is directly proportional to the volume involved in diffraction [31]. Thus, a large diffraction peak area corresponds to a large lattice area and a high lattice content. During the 4 h hydrogen charging experiment (in Figure 10a), the hydrogen concentration in the specimen peaks, resulting in the maximum diffraction peak. The penetration of hydrogen atoms enlarges the internal lattice of the Q690 high-strength steel weldments, causing the ferrite to expand under the influence of hydrogen. As the hydrogen charging time increases, the internal lattice size and diffraction peak value increase accordingly. Based on the mechanism of hydrogen-enhanced localized plasticity (HELP), solute hydrogen in the material influences dislocation motion by reducing the density of dislocations and altering elastic interactions with precipitates. This interaction results in an increased dislocation rate and a suppression of cross-slip [32].

The microstrain and dislocation density changes in the HAZ of the Q690 high-strength steel welded specimens before and after hydrogen charging are calculated via the WH method, as shown in Table 7, Table 8 and Table 9.

The microstrain in the HAZ increases from 9.75 × 10^−3^ to 1.69 × 10^−2^ after hydrogen charging time (4 h), and the dislocation density increases from 8.16 × 10^15^ to 2.43 × 10^16^ (in Table 7). The microstrain in the HAZ increases from 9.75 × 10^−3^ to 1.88 × 10^−2^ with hydrogen charging current density (90 mA/cm^2^), and the dislocation density increases from 8.16×10^15^ to 3.15 × 10^16^ (in Table 8). The microstrain in the HAZ increases from 9.75 × 10^−3^ to 2.69 × 10^−2^ after hydrogen charging solution concentration (10 g/L), and the dislocation density increases from 8.16 × 10^15^ to 6.75 × 10^16^ (in Table 9). The heightened microstrain and dislocation density contribute to increased spacing between lattice boundaries in the HAZ of the high-strength steel weldments, thereby compromising material integrity, reducing elongation, and increasing susceptibility to hydrogen embrittlement.

**Figure 11:**
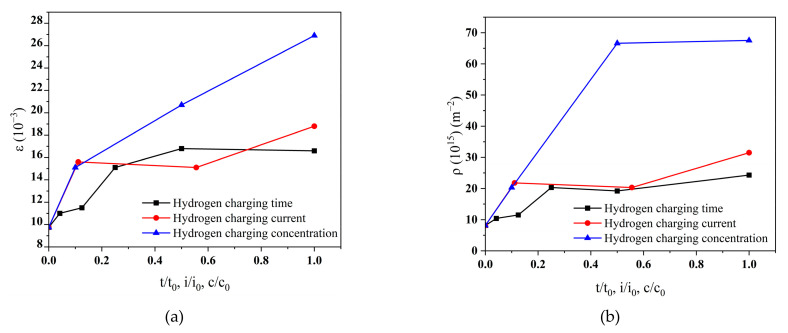
The microstrain and dislocation density vs. the normalized hydrogen charging parameters in the HAZ of Q690 weldments under different hydrogen charging conditions. (**a**) Microstrain and (**b**) dislocation density.

The relationship between the microstrain/dislocation density in the HAZ and the normalized hydrogen charging parameters are shown in Figure 11.

The microstrain and dislocation density in the HAZ increase with an increase in hydrogen charging parameters, with a large rate of growth in the initial stage of hydrogen charging which then tends to stabilize. The results indicate that the microstrain and dislocation density in the HAZ increase slowly when the hydrogen charging time and current density increase, but the effect of the solution concentration on the microstrain and dislocation density in the HAZ is significant.

### 3.3. Effect of Hydrogen Charging Conditions on the Fracture Morphologies of the HAZ

**Figure 12:**
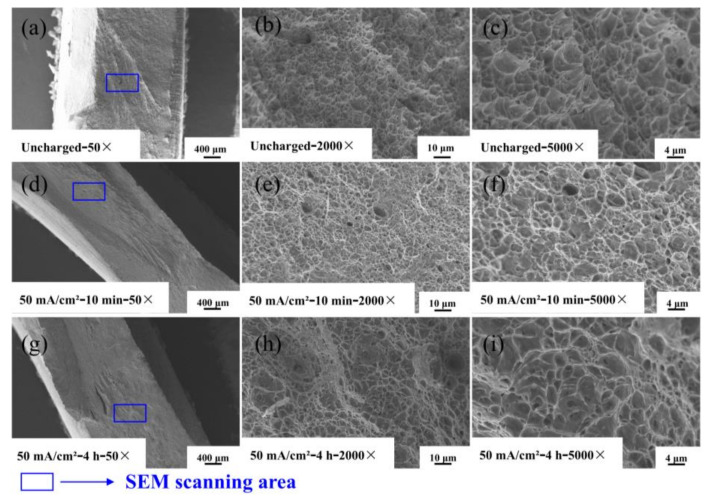
SEM map of fracture morphology of Q690 high-strength steel welded parts under different hydrogen charging times. (**a**–**c**) Uncharged with 50×, 2000×, and 5000×; (**d**–**f**) 50 mA/cm^2^—10 min with 50×, 2000×, and 5000×; (**g**–**i**) 50 mA/cm^2^—4 h with 50×, 2000×, and 5000×.

The fracture morphology of the experimental specimens at different hydrogen charging times are shown in Figure 12, from which it can be seen that the fracture areas of the samples are located in the heat-affected zone.

The first to third columns of the figure show the SEM images at 50×, 2000×, and 5000× magnifications, respectively. Figure 12a show that the fracture of Q690 high-strength steel weldments before hydrogen charging shows necking phenomenon and exhibits obvious fracture toughness characteristics, with many tiny particles adhered to the fracture surface. The corresponding high-magnification image shows the uniformly distributed toughness nests on the fracture surface. The white ridges surrounding the tough nests represent tearing prongs at the grain boundaries, which are indicative of typical ductile fractures. As the hydrogen charging time increases, Figure 12a,d,g show that the fracture surface gradually transitions to flat and smooth. This transformation is attributed to the penetration of hydrogen atoms, which reduces the elongation of the material and causes its fracture mode to shift from plastic fracture to hydrogen-induced brittle fracture. Pitting corrosion typically manifests in localized areas of a metal surface, resulting in small, deep etch holes. While it predominantly occurs in these localized regions, the relationship between pit diameter and depth can be complex. Notably, pitting corrosion is more likely to arise in concealed locations, such as crevices and cracks [31]. The microstructure results illustrate that with increasing hydrogen charge, the specimen’s surface becomes rougher and exhibits crack formation, thereby increasing the likelihood of pitting corrosion. This deformation results in the development of holes and cracks on the fracture surface [33].

**Figure 13:**
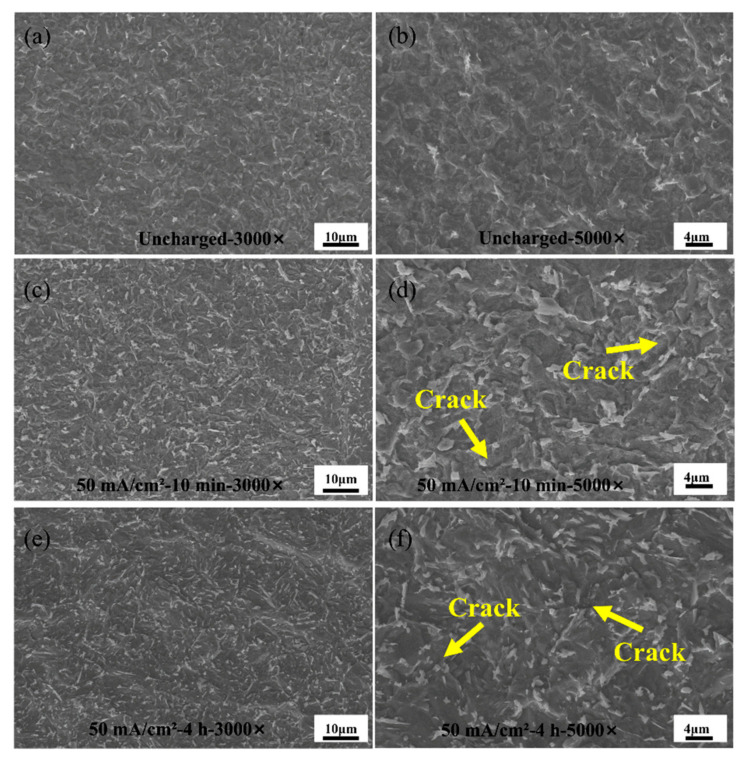
Characterization of the heat-affected zone of Q690 high-strength steel weldments for different hydrogen charging times. (**a**,**b**) Uncharged with 3000× and 5000×; (**c**,**d**) 50 mA/cm^2^—10 min with 3000× and 5000×; (**e**,**f**) 50 mA/cm^2^—4 h with 3000× and 5000×.

Hydrogen embrittlement is a phenomenon characterized by the penetration of hydrogen atoms into the steel surface, resulting in localized plastic deformations at defects or stress centres on the surface. Figure 13 shows the microstructural changes in the HAZ of Q690 high-strength steel weldments after different hydrogen charging times.

In the welding process, because the weldments’ base material area and fusion zone are a certain distance from each other, the influence of the high heat of the fusion zone on the base material area is small, so the base material area of the microstructure is more stable in the hydrogen charging experiments in which the hydrogen molecules’ penetration have the least influence on the HAZ. Figure 13a,b show that the surface of the HAZ of the specimen appears relatively smooth and flat when the specimen is not hydrogen-charged. The precipitation of carbides within the material occurs as a result of the temperature increase during the welding process. During electrochemical hydrogen charging, hydrogen atoms initially adhere to the material’s surface, leading to gradual damage over time to high-strength steel. As illustrated in Figure 13c,d, after 10 min of hydrogen charging, the material’s surface becomes rough, and cracks develop due to the accumulation of hydrogen atoms [34]. At 4 h of hydrogen charging time, as shown in Figure 13e,f, the surface of the experimental steel shows obvious hydrogenation defects and the carbides begin to refine. The penetration of hydrogen atoms on the material surface disrupts the original internal microstructure, resulting in a concave–convex morphology. The ingress of hydrogen disrupts the internal microstructure arrangement, increasing the levels of internal stress (tension) and tissue deformation [35]. This phenomenon aligns with the previous discussion on the reductions in mechanical properties, including tensile strength, yield strength, and elongation.

**Figure 14:**
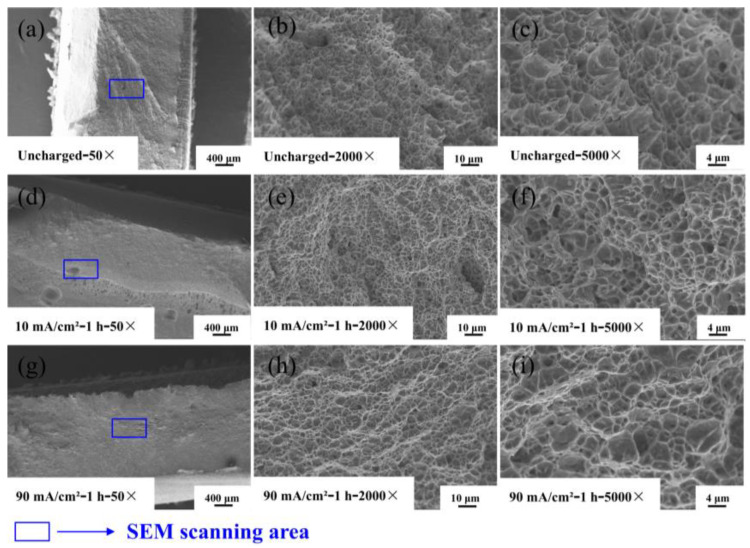
SEM maps of the fracture morphologies in the HAZ of Q690 high-strength steel welded parts with different hydrogen charging current densities. (**a**–**c**) Uncharged with 50×, 2000×, and 5000×; (**d**–**f**) 10 mA/cm^2^—1 h with 50×, 2000×, and 5000×; (**g**–**i**) 90 mA/cm^2^—1 h with 50×, 2000×, and 5000×.

The fracture topography of the heat-affected zone observed at different hydrogen charging current densities are illustrated in Figure 14. The micrographs of the fractures in Figure 14a,d,g reveal gradual transitions to relatively smooth and flat fracture surfaces in the welded parts of the Q690 high-tensile steels.

The holes and grooves appearing on the fracture surfaces in Figure 14d result from incomplete welding of the specimens during hydrogen charging experiments. When the current density is increased up to 10 mA/cm^2^, the fracture surface of high-strength steels develops hydrogenation pits of varying sizes, which can be observed as tough nest features of varying sizes when photographed with a high-magnification camera. These phenomena are attributed to the uncoordinated plastic deformation of ferrite and martensite within the material, resulting in a decrease in toughness [36]. Upon increasing the current density to 90 mA/cm^2^, brittle fracture characteristics become increasingly prominent, with the fracture surface appearing to be crystalline and flush, with a distinct metallic lustre. Microscopically, the fracture morphology reveals a tough fossa-like deconstructed structure characterized by a deconstructed tough fossa and tongue-like patterns. With increasing current density, the tough fossa size significantly increases, and the ridges near the tough fossa decrease in depth. This finding indicates that the material undergoes minimal plastic deformation, the specimen plasticity and elongation decrease, and brittle fracturing in the material is complete.

**Figure 15:**
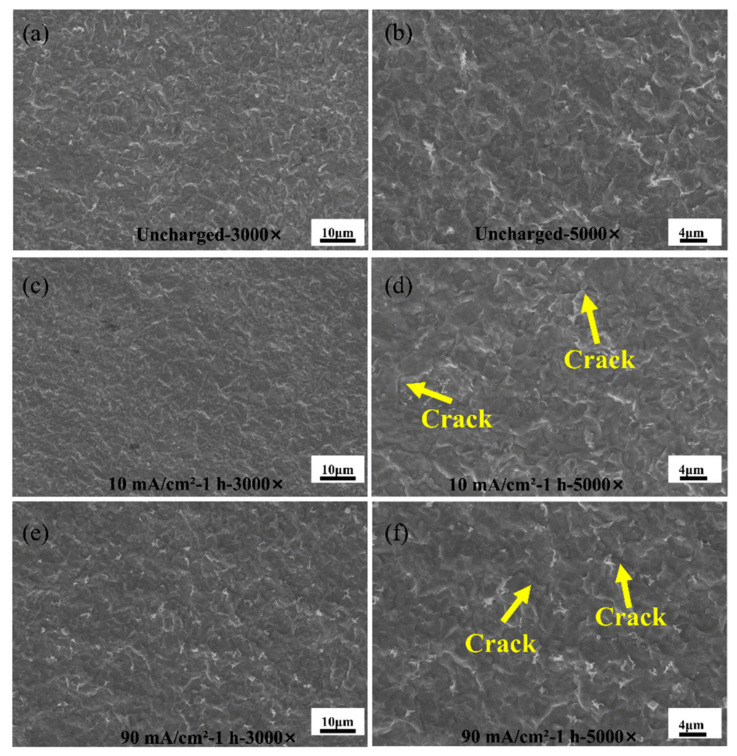
SEM microstructures in the HAZ of Q690 high-strength steel weldments with different hydrogen charging current densities. (**a**,**b**) Uncharged with 3000× and 5000×; (**c**,**d**) 10 mA/cm^2^—1 h with 3000× and 5000×; (**e**,**f**) 90 mA/cm^2^—1 h with 3000× and 5000×.

Figure 15 shows the microstructure of the HAZ of a welded part made of Q690 high-strength steel.

Evidently, the overall surface characteristics of the base material area change with increasing current density. With increasing hydrogen charging current, shallow hydrogen embrittlement cracks emerge on the surface of the base material. In addition, the infiltration of hydrogen molecules can lead to hydrogen-induced damage cracking [37]. The white carbide in the figure is due to the sudden increase in temperature during welding [38]. As the current density increases to 90 mA/cm^2^, the maximum hydrogen concentration within the material is reached, atomic hydrogen reconstitutes into gaseous hydrogen at hydrogen traps (including material defects such as inclusions, grain boundaries, dislocations, and hard-phase compositions), and further migration is inhibited. This phenomenon results in the formation of high localized pressures in the material [39]. Subsequently, crack propagation occurs as the internal pressure gradually increases. Moreover, the lattice area expands as hydrogen reaches its maximum concentration within the material, leading to the transformation of carburite from its initial cloud-like shape to an elongated leaf–stem-like configuration.

**Figure 16:**
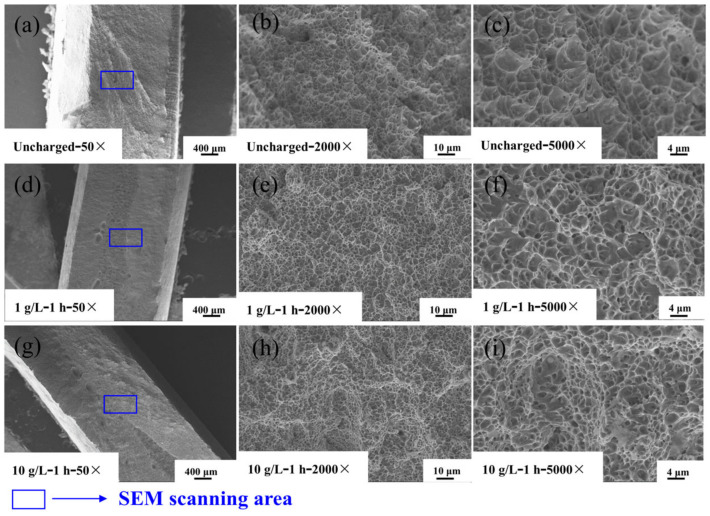
SEM map of fracture morphology in the HAZ of Q690 high-strength steel welded parts with different hydrogen solution concentrations. (**a**–**c**) Uncharged with 50×, 2000×, and 5000×; (**d**–**f**) 1 g/L—1 h with 50×, 2000×, and 5000×; (**g**–**i**) 10 g/L—1 h with 50×, 2000×, and 5000×.

Figure 16 shows the fracture morphology in the heat-affected zone of Q690 high-strength steel weldments and after 1 h of hydrogen charging at different solution concentrations.

As the concentration of the hydrogen-charging solution increases, the hydrogen content in the material significantly increases, exacerbating the corresponding hydrogen-induced damage. Surface stresses emerge as hydrogen accumulates within the material, and when the stress surpasses the yield strength of the material, deformation-induced bubbles and cracks may form [40]. With further increases in the hydrogen concentration after the material exhibits atomic cohesion, cracks begin to propagate on the surface, causing a transition from ductile to brittle fracture in the experimental steel. Consequently, hydrogen cracks and craters on the surface become increasingly abundant, exhibiting more pronounced brittle fracture characteristics with higher concentrations of the hydrogen-charging solution. Macroscopically, an increase in solution concentration results in the appearance of cracks of varying sizes on the fracture surface, rendering it relatively flat. Microscopically, many chicken-claw patterns and, to a reduced extent, fish-bone patterns of deconstructed fracture morphologies become visible. Under high magnification levels, black craters surrounding the fracture ligament fossa indicate that hydrogen penetration changes the internal microstructural arrangement of the material. The fracture ligament fossa densifies, and its size increases, thereby diminishing the plasticity of the material. Despite the fracture mode remaining deconstructive, the macroscopic fracture surface appears flush and smooth, while the microscopic fracture morphology exhibits scattered chicken-claw patterns. These patterns are indicative of brittle fracture.

**Figure 17:**
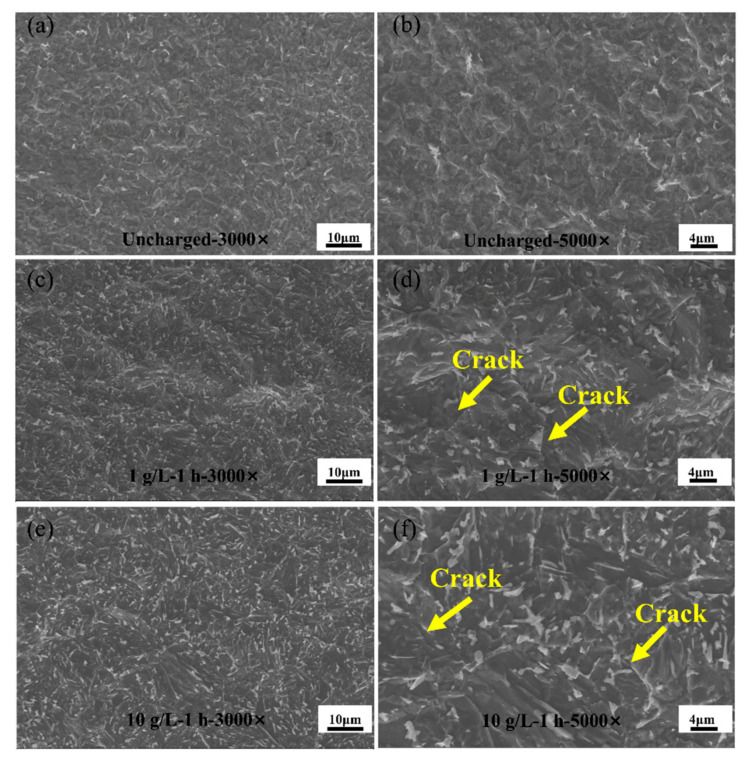
SEM microstructure of Q690 high-strength steel weldments in the heat-affected zone at different hydrogen solution concentrations. (**a**,**b**) Uncharged with 3000× and 5000×; (**c**,**d**) 1 g/L—1 h with 3000× and 5000×; (**e**,**f**) 10 g/L—1 h with 3000× and 5000×.

Figure 17 shows the microscopic characteristics of Q690 high-strength steel weldments at different concentrations in the HAZ.

Figure 17b,d,f show that hydrogen atoms have a significant effect on the corrosion penetration ability of high-strength steels in the base material zone. The cloudy white carbide observed on the surface of the experimental steel results from the nonuniform decomposition of heat during the transformation of ferrite to martensite during welding. With increasing solution concentration, hydrogen gradually penetrates the surface of the experimental steel, leading to the transformation of cloudy carbides into particles.

According to the above results, it is found that the failure of Q690 high-strength structural steel welded parts mainly occurs in the HAZ. The arrangement of ductile dimples on the fracture surface of the original sample is regular, and the shape of the dimples is an equiaxed ductile fracture surface. With the amount of hydrogen entering increasing, the fracture mode shifts from the ductile to the brittle, and the corresponding fracture surface gradually changes from irregular to flat. Non-uniform distribution of toughness dimples appears on the fracture surface, and the depth becomes shallower until it finally disappears. The surface of the fracture shows pits that gradually deepen and enlarge, and finally, the microscopic morphology of the fracture shows chicken-claw patterns spreading out to form a brittle fracture. At that time, the carbides precipitate and gradually aggregate into blocks in the fracture surface.

Hydrogen absorption in metals can significantly impair their mechanical properties, potentially leading to severe industrial accidents. Therefore, protecting metallic materials from hydrogen embrittlement is crucial. The heat treatment of welded joints can reduce hydrogen uptake in metals [41]. Applying coatings to metallic surfaces creates a barrier that inhibits hydrogen penetration, thereby limiting diffusion and reducing susceptibility to hydrogen embrittlement (HE) [42,43,44]. However, it is essential to address potential defects in the coating and evaluate the plasticity and bond strength between the substrate and the coating. Additionally, introducing residual compressive stresses at the surface can mitigate hydrogen embrittlement by enhancing the internal lattice structure and preventing hydrogen penetration [45].

## 4. Conclusions

In this study, the effects of hydrogen charging conditions on the macroscopic mechanical behaviours of Q690 high-strength steel weldments and the corresponding microscopic structures were investigated by using electrochemical hydrogen charging tests, SEM analyses, XRD analyses, and axial stretching methods. The research results can be summarized as follows:

1. The mechanical parameters (such as the tensile strength, yield strength, and elongation) of the Q690 high-strength steel weldments decrease nonlinearly with increasing hydrogen penetration. The decreasing rate of the tensile strength and yield stress is much lower than that of the elongation. The same is true of the variation in the residual factors related to the tensile strength. Initially, the duration of hydrogen charging solution concentration significantly affects the degradation of mechanical parameters. However, in the middle and later stages, the hydrogen charging time has a more pronounced influence on the degradation of mechanical parameters.
2. The microstrain and dislocation density in the HAZ increase with an increase in hydrogen charging parameters, with a large rate of growth in the initial stage of hydrogen charging, and then they tend to stabilize. The effect of the solution concentration is more significant than the hydrogen charging time and current density.
3. The arrangement of ductile dimples on the fracture surface of the original sample is regular, and the shape of the dimples is an equiaxed ductile fracture surface. With an increase in the amount of hydrogen entering, the fracture mode shifts from the ductile to the brittle, and the corresponding fracture surface gradually changes from irregular to flat. The surface of the fracture shows pits that gradually deepen and enlarge, and finally, the microscopic morphology of the fracture shows chicken-claw patterns spreading out to form a brittle fracture.

In the future, we plan to further investigate the mechanisms by which hydrogen atoms affect the multiscale mechanical properties of interfaces in high-strength steel and the durability of structures exposed to hydrogen.

## Figures and Tables

**Table 2:** Chemical composition table of welding wire (mass fraction %).

Chem. Comp.	C	Mn	Si	S	P	Cr	Ni	Mo	Cu
value	≤0.11	1.40~1.85	0.40~1.00	≤0.025	≤0.025	0.25~0.60	1.20~2.40	0.20~0.60	≤0.50

**Table 3:** Hydrogen charging experiment program.

No.	Charging Time (h)	Current Density (mA/cm^2^)	Concentration Solution (g/L)
1	1/6, 1/2, 1, 2, 4	50	1
2	1	10, 50, 90	1
3	1	50	1, 5, 10

**Table 4:** Mechanical parameters and corresponding residual coefficients of welded parts of Q690 high-strength steel with different hydrogen charging times.

Time	*σ_b_* (MPa)	*σ_b_*/*σ_b_*_0_	*σ_s_* (MPa)	*σ_s_*/*σ_s_*_0_	*δ* (%)	*δ*/*δ*_0_
Uncharged	839.59	1	772.63	1	30.40	1
10 min	836.43	0.996	770.54	0.997	28.80	0.947
30 min	833.00	0.992	765.71	0.991	28.00	0.921
1 h	827.48	0.986	760.10	0.984	26.52	0.872
2 h	812.35	0.968	748.61	0.969	25.60	0.842
4 h	805.66	0.960	741.40	0.960	24.48	0.805

**Table 5:** Mechanical parameters and corresponding residual coefficients of Q690 high-strength steel weldments at different current densities.

Current Density	*σ_b_* (MPa)	*σ_b_*/*σ_b_*_0_	*σ_s_* (MPa)	*σ_s_*/*σ_s_*_0_	*δ* (%)	*δ*/*δ*_0_
Uncharged	839.59	1	772.63	1	30.40	1
10 mA/cm^2^	836.58	0.996	767.11	0.993	28.92	0.951
50 mA/cm^2^	827.48	0.986	760.10	0.986	26.52	0.872
90 mA/cm^2^	825.56	0.983	752.46	0.974	25.20	0.829

**Table 6:** Mechanical parameters and corresponding residual coefficients of welded parts of Q690 high-strength steel at different solution concentrations.

Solution Concentration	*σ_b_* (MPa)	*σ_b_*/*σ_b_*_0_	*σ_s_* (MPa)	*σ_s_*/*σ_s_*_0_	*δ* (%)	*δ*/*δ*_0_
Uncharged	839.59	1	772.63	1	30.40	1
1 g/L	827.48	0.986	760.10	0.984	26.52	0.872
5 g/L	824.27	0.982	757.77	0.981	25.64	0.843
10 g/L	820.39	0.977	746.90	0.967	24.20	0.796

**Table 7:** Microstrain and dislocation density in the HAZ of materials under different hydrogen charging times.

Hydrogen Charging Time	Parameters	(110)	(200)	(211)	Total
Uncharged	ε	4.52 × 10^−3^	2.02 × 10^−3^	3.20 × 10^−3^	9.75 × 10^−3^
	ρ (m^−2^)	9.52 × 10^15^	2.42 × 10^15^	4.80 × 10^15^	8.16 × 10^15^
10 min	ε	5.14 × 10^−3^	2.22 × 10^−3^	3.60 × 10^−3^	1.10 × 10^−2^
	ρ (m^−2^)	6.20 × 10^15^	1.16 × 10^15^	3.03 × 10^15^	1.04 × 10^16^
30 min	ε	5.45 × 10^−3^	2.29 × 10^−3^	3.76 × 10^−3^	1.15 × 10^−2^
	ρ (m^−2^)	6.96 × 10^15^	1.23 × 10^15^	3.31 × 10^15^	1.15 × 10^16^
1 h	ε	7.42 × 10^−3^	2.55 × 10^−3^	4.27 × 10^−3^	1.51 × 10^−2^
	ρ (m^−2^)	4.28 × 10^15^	1.52 × 10^15^	1.29 × 10^16^	2.03 × 10^16^
2 h	ε	7.08 × 10^−3^	2.70 × 10^−3^	4.68 × 10^−3^	1.66 × 10^−2^
	ρ (m^−2^)	1.18 × 10^16^	1.71 × 10^15^	5.12 × 10^15^	1.92 × 10^16^
4 h	ε	8.05 × 10^−3^	3.15 × 10^−3^	5.37 × 10^−3^	1.69 × 10^−2^
	ρ (m^−2^)	1.52 × 10^16^	2.33 × 10^15^	6.76 × 10^15^	2.43 × 10^−6^

**Table 8:** Microstrain and dislocation density in the HAZ of materials under different hydrogen charging current densities.

Current Density	Parameters	(110)	(200)	(211)	Total
Uncharged	ε	4.52 × 10^−3^	2.02 × 10^−3^	3.20 × 10^−3^	9.75 × 10^−3^
	ρ (m^−2^)	9.52 × 10^15^	2.42 × 10^15^	4.80 × 10^15^	8.16 × 10^15^
10 mA/cm^2^	ε	7.71 × 10^−3^	2.87 × 10^−3^	5.02 × 10^−3^	1.56 × 10^−2^
	ρ (m^−2^)	1.39 × 10^16^	1.93 × 10^15^	5.90 × 10^15^	2.18 × 10^16^
50 mA/cm^2^	ε	7.42 × 10^−3^	2.80 × 10^−3^	4.87 × 10^−3^	1.51 × 10^−2^
	ρ (m^−2^)	1.29 × 10^16^	1.84 × 10^15^	5.55 × 10^15^	2.03 × 10^16^
90 mA/cm^2^	ε	9.31 × 10^−3^	3.42 × 10^−3^	6.03 × 10^−3^	1.88 × 10^−2^
	ρ (m^−2^)	2.02 × 10^16^	2.74 × 10^15^	8.52 × 10^16^	3.15 × 10^16^

**Table 9:** Microstrain and dislocation density in the HAZ of materials under different hydrogen charging solution concentrations.

Solution Concentration	Parameters	(110)	(200)	(211)	Total
Uncharged	ε	4.52 × 10^−3^	2.02 × 10^−3^	3.27 × 10^−3^	9.75 × 10^−3^
	ρ (m^−2^)	9.52 × 10^15^	2.42 × 10^15^	4.80 × 10^15^	8.16 × 10^15^
1 g/L	ε	7.42 × 10^−3^	2.55 × 10^−3^	4.27 × 10^−3^	1.51 × 10^−2^
	ρ (m^−2^)	1.29 × 10^16^	1.52 × 10^15^	4.28 × 10^15^	2.03 × 10^16^
5 g/L	ε	1.04 × 10^−2^	3.65 × 10^−3^	6.60 × 10^−3^	2.07 × 10^−2^
	ρ (m^−2^)	2.52 × 10^16^	3.12 × 10^15^	1.02 × 10^16^	6.66 × 10^16^
10 g/L	ε	1.40 × 10^−2^	4.43 × 10^−3^	8.50 × 10^−3^	2.69 × 10^−2^
	ρ (m^−2^)	4.60 × 10^16^	4.59 × 10^15^	1.69 × 10^16^	6.75 × 10^16^

## Data Availability

Data are contained within the article.
